# The role of midwifery associations in the professional development of midwifery

**DOI:** 10.18332/ejm/122388

**Published:** 2020-07-02

**Authors:** Serap Ö. Altınayak, Serap E. Apay, Joeri Vermeulen

**Affiliations:** 1 Department of Midwifery, Faculty of Health Science, Ondokuz Mayıs University, Samsun, Turkey; 2 Department of Midwifery, Faculty of Health Science, Atatürk University, Erzurum, Turkey; 3 Department Health Care, Knowledge Centre Brussels Integrated Care, Erasmus Brussels University of Applied Sciences and Arts, Brussels, Belgium; 4 Department of Public Health, Biostatistics and Medical Informatics Research group, Faculty of Medicine and Pharmacy, Vrije Universiteit Brussel, Brussels, Belgium

**Keywords:** associations, midwifery associations, midwifery associations in Turkey, midwifery associations in the world

## Abstract

Midwifery associations have an important role in several subjects such as professionalisation, developing neonatal health, reducing maternal-fetal mortality, planning labor, professional recognition, planning legislative regulations, developing the profession, and offering quality birth services. The purpose of this study is to explain the contributions of well-known midwifery associations in the world, and in particular in Turkey, to the professional development of midwifery, as well as similarities of midwifery associations in Turkey with other associations, including the position of Turkey in associationalism. The associations have enabled the profession of midwifery to progress, gain autonomy, be legally protected and become more professional. In order for the associations in Turkey to operate effectively, it is recommended that their membership is increased so as to represent more midwives and thus gain more political power and to advance evidence-based applications in midwifery care.

## COMMENTARY

A midwifery association is defined as a platform for developing strong, supportive, positive relationships among midwives and between the profession of midwifery and other stakeholders such as governments and other health professionals^1^. Midwives alone may not be sufficient to correct the negativities in professional practices, to fulfil the requirements of the age, or to gain advances socially about the profession of midwifery^2^. By contributing professional experience and expertise, professional associations may play a significant role in informing and influencing reproductive, maternal, and child health policy and practice^3^. Midwives need professional support from midwifery associations to be able to provide the services that are, by regulatory mechanisms and accreditation, expected of them^4^. The midwifery community can only achieve this aim by focusing on a specific purpose related to the midwifery profession with an association.

International publications identify midwifery as important for improving maternity care worldwide. Midwifery is a team effort where midwives play a key role^5^. Midwives’ involvement in maternity care, as the health professional of first choice, is fundamental to maternal and neonatal health^5,6^. The World Health Organization (WHO) and partners including the International Confederation of Midwives (ICM) and the United Nations Population Fund (UNFPA) recently highlighted the challenging conditions midwives often face, and advocated for increased investment in midwifery^7^. For this purpose, it is necessary to increase the number of qualified and competent midwives^8^. As a result of this requirement, a joint initiative for the global action on midwifery has been organised in 2010 with the participation of a broad coalition formed by maternal and child health organisations including ICM, UNFPA, WHO, and the International Federation of Gynecology and Obstetrics (FIGO). With this meeting, governments have stated the need to address vital areas that are the basis for a strong midwifery profession such as midwifery associations, midwifery education, regulation, and research. According to Bogren et al.^9^, UNFPA expressed in 2011 in the World’s Midwifery Report, one year after the meeting, that ‘if one of these areas is weak, the whole midwifery will be weak’. With this report, UNFPA has once again stated the importance of professional midwifery associations to develop.

Midwifery associations can play a vital role in many subjects such as the development of mother-child health, reduction of maternal-fetal mortality, and enhancing maternal-fetal and neonatal health, worldwide. Professional associations are important partners in national policy discussions and decisions about maternal and newborn health^1^. Associations should contribute to policy development together with governments in order to ensure the sustainability of maternal and neonatal health. However, most of the associations stated that they could not participate directly in decisions about policy making and they could only participate in the process indirectly^2^. In this context, roles of professional associations are being involved in the process of direct policy-making, raising awareness, forming a team, setting practice standards, and establishing lobbies on women’s health and rights. Besides, professional associations in the developed countries can influence and strengthen their colleagues in the developing countries, e.g. through twinning projects^10^. Twinning is a cross-cultural, reciprocal process where two professional associations work together to achieve joint goals^11^. Professional associations may encourage them to play an active role in reducing maternal mortality abroad and in their own countries^10,12^. Especially in countries where maternal-fetal mortality has become an epidemic, there is a need for organisations such as midwifery associations to support reducing this problem. Moreover, the warnings of midwifery associations are even more important in promoting changes in these countries. Thus, the increase of the recognition of the associations will probably increase their capacity of being effective^1^. Midwifery organisations have the potential to initiate structural changes in maternity care, which will facilitate a more women-centered care^13^. As is seen, midwifery associations play a crucial role in several areas, such as reducing maternal-fetal mortality, promoting newborn’s health and providing midwifery education, professional progress, and professionalisation. Thus, this study was conducted for the purpose of explaining the role of associations, how they operate, contributions of well-known midwifery associations in the world, and in Turkey, to the professional development of midwifery, as well as similarities of midwifery associations in Turkey with these associations, and the position of Turkey in associationalism, i.e. in the process of forming an association. In addition, another aim of this study was to provide the reader with a summary about midwifery associations.

### The roles and duties of midwifery associations

Midwifery associations have many roles, which they can adapt both in developed and developing countries. Besides, each can improve the health of mother and newborn in terms of many aspects such as reducing maternal-fetal mortalities and improving health. To provide these, midwifery associations can provide infrastructure with its role of education advocacy, ensure human/health resources to access adequate education level, determine health care agenda politically by establishing political lobbies, ensure the allocation of financial resources in order to support this process economically, and provide public awareness and social knowledge by organising awareness campaigns^10^.

**Figure 1:**
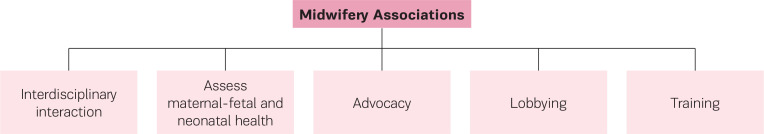
Active/potential interventions of midwifery associations

Midwifery associations can raise awareness for the maternal and infant health by lobbying and making advocacy on the principles of effective health services for women and newborns at the level of the public, governments, and international aid organisations, ensuring interdisciplinary professional interaction, assessing maternal-fetal and neonatal health, and educating the midwives and the society to which they provide services (Figure 1). These associations promote collaboration between perinatal health professionals and also can be strong service providers to women^10^. Associations have the potential to make a difference by influencing the health services provided to women in their own countries and other professional associations in other countries. The most well-known international and Turkish midwifery associations, and their aims and activities, are described below.

### Selected international midwifery associations

#### International Confederation of Midwives (ICM)

ICM is as an accredited non-governmental organisation representing midwives from associations all over the world aiming to achieve common goals in maternal and neonatal care^14^. The ICM was founded in the early 1900s. Initially ICM organised several international meetings with midwives in the European continent only. However, ICM was not able to organise any meeting during an eight-year period (1914–1922) due to the First World War. Currently, the ICM represents 132 midwifery associations and 500000 midwives from 113 countries in each continent (Table 1). ICM is organised in six regions: Africa, America, Western Pacific, Eastern Mediterranean, Southeast Asia, and Europe^14^. ICM cooperates closely with organisations such as: WHO, UNFPA, FIGO, European Midwives Association (EMA), International Council of Nurses (ICN), and the International Pediatric Association (IPA). The ICM aims to strengthen midwifery associations and the midwifery profession, to improve the reproductive health of women and newborns, and encourages the development of autonomous midwifery^15^. In order to take the midwifery profession to an advanced stage, ICM participates in discussions about the future of midwifery education, to develop various projects and apply knowledge for the protection of women’s fundamental rights. The ICM advocates that midwives should be in a position protecting justice, trust, security, dignity, and autonomy values, and ensuring safe birth practices for women, providing holistic care, and making an effort to develop midwifery education^16^. Besides, ICM underlines the crucial role of continuous professional development on the maintenance of safe and current practice in maternity care. The member associations in the scope of ICM also lead to constant educational updates in line with the international code of ethics for midwives^10^. In this context, ICM appears as an organisation aiming to guide the education, practice, and research of midwives in accordance with the aim of focusing on women’s health and midwives, and to develop the standards of care allowing the improvement and post-graduate education of midwives^17^. Turkey has been a member since 2011 without interruption.

**Table 1:** Figures and facts of the studied international midwifery associations

*Associations*	*Headquarters location*	*Established*	*Qualifications for membership*	*Membership*	*The Association’s role*		
					*Regulation*	*Research*	*Education (including CPD)*
**International Confederation of Midwives**	The Netherlands	1919	Midwifery associations worldwide	132 associations from 113 countries, 500000 members	X	X	X
**Royal College of Midwives**	United Kingdom	1881	Midwives, Student midwives, Maternity support workers	47000 members	X	X	X
**Midwives Alliance of North America**	United States of America	1982	Midwives, Student midwives, Midwifery support workers	500 members	X	X	X
**National Association of Certified Professional Midwives**	United States of America	1995	Midwives, Student midwife	380 members	X		X
**American College of Nurse-Midwives**	United States of America	1955	Certified midwives, Student midwives, Certified nurse-midwives, Retired midwives, Industry partners (Healthcare companies) Associates (Individuals who promote and/or retailers of midwifery and women’s health products)	6500 members	X	X	X
**European Midwives Association**	Belgium	1968	Midwifery associations from the European Union, the European Economic Area and from the Council of Europe	37 associations from 30 countries	X	X	X

#### Royal College of Midwives (RCM)

The Royal College of Midwives was established in 1881 under the name ‘Trained Midwives Registration’ Society (Table 1). However, it continues to exist with its current name since 1947. RCM is the leading professional association in England supporting midwives. It was established for the purpose of improving and protecting professional midwifery standards. The RCM organises many activities such as workforce planning and conducting various research, and organising professional development courses, conferences, and campaigns. RCM has offices in Scotland, Northern Ireland, Wales, and the United Kingdom^18^. The organisation has basically two tasks: being a professional midwifery association, and a union that protects the rights of midwives through legal processes. The RCM carries out tasks such as ensuring the advancement of the midwifery profession and cooperating with the relevant organisations, health professionals and the government. The primary purpose of RCM is to bring midwifery education to a high level in all fields, increase the quality of midwifery education and care, make necessary legal arrangements, and establish strong lobbies to reach specific targets^19^.

Within the scope of midwifery, RCM focuses on subjects such as providing high quality care to women, newborns and their families, encouraging midwives to apply professional standards, supporting its members, representing the interests of the parents individually and collectively, and encouraging innovation and leadership in maternity care. It is an organisation trying to increase the midwives’ trust and professional practices for the benefit of the women and their families. The RCM, through all these components, aims to ensure that midwives work in honesty, act in an open and transparent manner, reach its members and provide equality of service^20^.

#### Midwives Alliance of North America (MANA)

MANA was founded in 1982 by midwives starting with the home birth movement in the 1970s in the USA, aiming to support normal birth and provide service to women who chose to give birth at home. The association aims to advance and develop the progress of midwifery. Besides, MANA also aims to design an innovative midwifery model aiming to provide access to women and family-oriented care. MANA developed the first national certification exam for midwives, and a national registry record was initiated in 1986. In this way, it established the foundation for the creation of Certified Professional Midwife IDs. By the early 1990s, MANA developed the Declaration of Values and Ethics for guiding professional behavior in midwifery practices. MANA values are explained by considering the mother and baby as a whole, supporting normal birth, and developing professional relations and midwifery practices, and considering that the mother and women are valuable^16,21,22^. Additionally, it defined the basic competencies, the assessment and clinical skills midwives need in the practice, and assessment documents for the professionalisation of midwives. In the early 1990s, MANA began collecting data about out-of-hospital births to assess midwifery practices conducted in North America. In 2004, MANA implemented a new online data collection system in order to continue data collection about out-of-hospital births^21^.

#### National Association of Certified Professional Midwives (NACPM)

National Association of Certified Professional Midwives (NACPM) is an organisation that specifically represents Certified Professional Midwives (CPM) in the USA. CPMs are a part of a long and versatile tradition of midwifery in the USA. They have a history based on encouraging home births in the early 1970s when women began to look for alternatives for the medicalisation of birth. The first certified professional midwives were accepted in 1995 with the organisation and professionalisation of these midwives. CPMs established NACPM in 2000 to provide a strong, unified voice and support active professional midwives^23^.

Certified Professional Midwives are knowledgeable and capable perinatal health professionals. Midwives have been trained and authorised to offer specialist care, education, counselling and support for women during pregnancy, childbirth and postpartum period. Besides, they are autonomous healthcare professionals who work in cooperation with other perinatal health professionals when needed. All Certified Professional Midwives must meet the certification standards set by the North American Registry of Midwives (NARM). In the USA, Certified Professional Midwives help to provide access to normal birth. Although they are authorised to practice in all settings, they have the special authority to provide care at home and in free-standing maternity centers and are currently working in more than half of the birth centers in the USA^24^. In 2004, NACPM established a document that sets a standard for practices and serves as an ethical code in order to define the practices and determine the core values, ethical principles, and philosophical approaches. It also supports the education of pregnant women in order to provide high-quality evidence-based maternal care and to encourage vaginal birth^16^.

#### American College of Nurse-Midwives (ACNM)

In the mid-1940s, a separate section was created for midwives and nurses under the National Organization of Public Health Nurses (NOPHN) established by public health nurses. In 1949, this organisation published the first national descriptive data on nurses and midwives. A few years later, when the national public health nurse organisation was reorganised, it included the American Nurses Association (ANA) and the National League for Nursing (NLN) for nurses. However, ACNM did not define and include the presence of nurse-midwives. In 1955, ACNM was established as a professional organisation for certified midwives and nurses (Table 1)^25^. ACNM aims to provide neonatal care, postpartum care, and vaginal birth, encourage women to optimal pregnancy, develop women and gynecological care and specialised midwifery practices, and support midwives. ACNM is an organisation supporting midwifery practice, encouraging institutional policies, regulations, fair and equitable laws, expanding the midwifery workforce, and encouraging advocacy, research and education as the care standard for women^26^. It is emphasised within the service philosophy of ACNM that the members should respect the dignity and rights of people, and get the best model for the health care of the women and their families. In addition, it also values the formal education, individual lifelong learning, conducting researches in line with ethical rules, and reflecting the results to midwifery practices in particular. It is an institution aiming to develop and maximise the health of women and families at the community, state, national and international level worldwide in line with these beliefs and values^16,27^.

#### European Midwives Association (EMA)

The Association was founded in 1968 under the name of European Midwives Liaison Committee (EMLA) but changed its name to the European Midwives Association (EMA) in 2004. The EMA is a non-profit association representing midwife organisations and associations from member countries of the European Union (EU) and the European Economic Area (EEA). The Association works in cooperation with other European professional organisations and non-governmental organisations to demonstrate the voice of midwives in Europe and to show the presence of midwives. As an umbrella organisation, EMA currently incorporates 37 midwifery organisations from 30 countries: 26 from the 28 EU countries, 2 from the European Economic Area (Iceland and Norway) and 2 from the Council of Europe (Switzerland and Turkey)^28^ (Table 1). In this way, it has a strong role in establishing communication with the policy makers and providing information and evidence at the national and European level by lobbying^6,29,30^. The Turkish midwifery association has been a member of EMA since 2016.

EMA has an important role in securing quality midwifery education and practice throughout Europe^28^ and provides a forum for discussing problems about midwives and women’s health for European midwives. Besides having the duties of protecting their presence and contribution in areas affecting the health policy and midwifery area in European Union (EU) and providing minimum standards for midwifery education and practices in EU, it is an association establishing connections with European and global health professional organizations^28^.

### Turkish midwifery associations

#### Turkish Midwives Association

Turkish Midwives Association was founded in 1954 by a group of six people within the body of the Istanbul University Cerrahpasa Medical Faculty under the presidency of Midwife Sekine Arcan in Istanbul (Table 2)^31^. The Association aims to strengthen the society, support and strengthen professional midwifery along with promoting and increasing the health of women, infant, family and community to take the midwifery profession to a proper scientific platform in accordance with the contemporary criteria, and to protect the professional rights and responsibilities of midwives^32^.The Association also took part in the development of midwifery education. The Association has an Istanbul branch. Graduates of health vocational high schools, all midwives with an associate, undergraduate or graduate degree, and nurses with a Master’s degree in the field of obstetrics and gynaecology can become members of the Association.

**Table 2:** Figures and facts of the studied Turkish midwifery associations

*Associations*	*Established*	*Qualifications for Membership*	*membership*	*The Association’s role*		
				*Regulation*	*Research*	*Education (including CPD)*
**Turkish Midwives Association**	1954	Midwives, Nurses with Master’s degree in Obstetrics and Gynecology	3757	X	X	X
**Midwives and Nurses Association**	2007	Midwives, Nurses, Health officers	5000	X		X
**Midwifery Education Research and Development Association**	2014	Midwifery, Maternity support workers	79		X	X
**Anatolian Midwives Association**	2018	Midwives, Maternity support workers	500	X		X

#### Midwives and Nurses Association (EHEMDER)

Founded in 2007, the Midwives and Nurses Association has been working on raising the social, economic and personal rights of midwives, especially the member portfolio of the officers in the health field and the midwives and nurses in other categories, to undergraduate and graduate-level European Union standards and norms, protecting and improving their rights, and supporting their activities^33^. Because the Association protects the rights of not only midwives but also nurses and health officers, it is dinstinct from other midwifery associations in Turkey.

#### Midwifery Education Research and Development Association (EBEARGE)

The Midwifery Education Research and Development Association was founded in 2014 (Table 2). The Association aims to enhance the quality of midwifery education, to conduct studies about the midwifery profession, and to promote the scientific development of the profession. The center of the Association is located in Izmir^34,35^. The Association aims to ensure the midwives in Turkey comply with current developments quickly, to support the research of educational midwives, to increase the number of research publications producing reliable information and innovations along with strengthening the quality of education, and to guarantee the quality of midwifery services^34^. The Association usually organises educational activities such as research, courses, seminars, conferences and panels for the development of the midwifery profession^35^. The distinctive feature of this Association from other Turkish associations is that its focus is generally on supporting midwifery education and research.

#### Anatolian Midwives Association (AMA)

The Anatolian Midwives Association was established in 2018; the headquarters of the Association are located in Ankara (Table 2). The Association aims to provide midwives with the necessary knowledge and skills and adequate equipment to enable them to carry out their profession under the current and legal regulations. To achieve this aim, the Association aimed to provide access to continuing education rights for the midwives to improve themselves and maintain their competence, to provide safe maternity care by promoting midwifery labour at the national level, and to protect professional autonomy and moral rights of midwifery. The Association plans to conduct research to develop the profession on a scientific basis, to organise meetings, to plan continuous development activities, to provide professional, scientific and social development opportunities in the field of birth and women’s health, and to provide effective and contemporary solutions at high standards to the personal, institutional and social needs of midwifery^36^.

## DISCUSSION

Many different associations have been established in the world, and in Turkey, for the development of the midwifery profession. Recent research revealed that in all 29 European countries, a professional organisation unique for midwives was identified, seven countries had more than one association for midwives, sometimes combined with nurses or other health care professionals. Seven countries stated they had a midwifery unit under the auspices of a larger nursing organization^28^. Considering the associations in the USA, it is seen that there are three associations established at different times, as in Turkey. Among these associations, the ACNM that was established in 1955 includes nurses and midwives, as does the Turkish EHEMDER that was established in 2007. Both associations work basically to enact laws and gain rights. As nurses in both countries are also members of these associations, they increase the membership and so the associations become politically more influential. In addition, among the associations established in the USA, the MANA (1982) and the NACPM (2000) are associations founded to basically promote home birth. The Turkish Association of Midwives established in 1954 and the Anatolian Midwives Association established in 2018 have similar goals. The increase in membership in the Anatolian Midwives Association in a relatively short time suggests that the Association can be considered a hope for the procurement of legislative regulations expected by midwives for years. On the other hand, the deep-rooted past of the Turkish Association of Midwives gives midwives institutional confidence. A similar situation can also be asserted for the USA. The presence of several associations in the USA and Turkey creates competition between the associations. Although competition between the associations may bring positive outcomes, like compelling the associations to work harder, the sharing of possible members weakens the associations both legally and politically.

There are associations in the USA such as ACNM, and in Turkey such as EHEMDER, where nurses and midwives are common members. Being a partner with other professional groups with different roots or part of these groups instead of advocating for a professional, unique culture and care philosophy in these associations, will create some challenges for the future^28^. Considering England, the deep-rooted past of the RCM, established in 1881, gives institutional confidence. Being another association having a long-standing background, ICM is an association with a high level of reliability that draws power from its deep roots. Similarly, another association protecting the rights of midwives in Europe and trying to raise their voice is EMA. In Turkey, the Turkish Midwives Association has a long-standing history. These four associations draw their power from not only their deep-rooted history but also their large membership. Maintaining a professional culture in such deep-rooted associations is important. Sustaining a professional culture is viewed as a responsibility of the professional associations^28^. Large member numbers make the associations politically stronger, enabling them to legalise midwifery practices and provide and protect the legal rights of midwives. The fact that an Association is established and administered by midwives in a country creates such a situation.

To be autonomous partners in maternity care, and a valuable resource for achieving optimum results, midwives need a strong profession in all countries in Europe. This study shows progress in the areas of education and academic research, however, it also highlights serious weaknesses and challenges in regard to midwives’ current roles in maternity care practice and legislation as well as their influence on health care culture and politics^1^.

## CONCLUSION

This study, with a focus on the current state of professional development in Turkey and its relevance to international midwifery associations, however, is not to be seen as a comprehensive evaluation of midwives associations at the international level. Professional associations are important for professionalisation, development and strengthening of a profession, also in midwifery. Many of the international midwifery associations, which are highlighted in this article have come a long way and have deep roots, compared to the midwifery associations in Turkey. It has been demonstrated in the literature that the number of years of existence and experience of professional associations is critical for successful engagement in the decision-making processes1. The advances in the international associations have led to the midwifery profession abroad to advance, gain autonomy, be taken under legal protection, and set on a more professional ground. The Turkish Midwives Association is the most established Association in Turkey, and it is seen that the newly founded associations aim to provide more professionalisation of the midwifery profession, to establish legal protection and autonomy in Turkey with academic work. If the associations in Turkey combine their forces under one association, they will have a stronger say and midwives will have more political power. In order for midwifery associations to function effectively, it is recommended that they represent more people by increasing their membership and so gain more political power and improve the quantity and quality of academic studies to increase evidence-based practices in midwifery care.
